# Optimal level of *purple acid phosphatase5* is required for maintaining complete resistance to *Pseudomonas syringae*

**DOI:** 10.3389/fpls.2015.00568

**Published:** 2015-08-04

**Authors:** Sridhar Ravichandran, Sophia L. Stone, Bernhard Benkel, Junzeng Zhang, Fabrice Berrue, Balakrishnan Prithiviraj

**Affiliations:** ^1^Department of Environmental Sciences, Faculty of Agriculture, Dalhousie UniversityTruro, NS, Canada; ^2^Department of Biology, Dalhousie UniversityHalifax, NS, Canada; ^3^Department of Plant and Animal Sciences, Faculty of Agriculture, Dalhousie UniversityTruro, NS, Canada; ^4^Aquatic and Crop Resource Development, National Research Council CanadaHalifax, NS, Canada; ^5^Department of Chemistry, University of Prince Edward IslandCharlottetown, PE, Canada

**Keywords:** *purple acid phosphatase5* (*PAP5*), *Pseudomonas syringae* pv. tomato DC3000, disease resistance, *Arabidopsis*, reactive oxygen species (ROS)

## Abstract

Plants possess an exceedingly complex innate immune system to defend against most pathogens. However, a relative proportion of the pathogens overcome host's innate immunity and impair plant growth and productivity. We previously showed that mutation in *purple acid phosphatase* (*PAP5*) lead to enhanced susceptibility of *Arabidopsis* to the bacterial pathogen *Pseudomonas syringae* pv. *tomato* DC3000 (*Pst* DC3000). Here, we report that an optimal level of *PAP5* is crucial for mounting complete basal resistance. Overexpression of *PAP5* impaired *ICS1, PR1* expression and salicylic acid (SA) accumulation similar to *pap5* knockout mutant plants. Moreover, plant overexpressing *PAP5* was impaired in H_2_O_2_ accumulation in response to *Pst* DC3000. *PAP5* is localized in to peroxisomes, a known site of generation of reactive oxygen species for activation of defense responses. Taken together, our results demonstrate that optimal levels of *PAP5* is required for mounting resistance against *Pst* DC3000 as both knockout and overexpression of *PAP5* lead to compromised basal resistance.

## Introduction

Plants are constantly exposed to a diverse array of microbial pathogens. In nature, plants have evolved mechanism(s) to restrict most pathogens (non-host disease resistance) and also reduce pathogen ingress (basal resistance or PAMP-triggered immunity) (Bittel and Robatzek, 2007). Activation of defense response begins with the recognition of conserved molecular signatures or PAMP (Pathogen-Associated Molecular Pattern) by pattern recognition receptors (PRRs) localized on the plasma membrane and in the cytoplasm (Chisholm et al., 2006; Jones and Dangl, 2006). PAMPs are microbial molecular signatures (e.g., flagellin, bacterial lipopolysaccharides, elongation factor, chitin, and β-glucan) that are absent in the host (Boller and Felix, 2009; Schwessinger and Ronald, 2012). Following PAMP perception, the PRRs initiate complex signaling networks that are associated with rapid synthesis of reactive oxygen species, activation MAP kinase signaling and pathogenesis related (PR) genes leading to PAMP triggered immunity (PTI) (Chisholm et al., 2006). However, well-adapted microbial pathogens have evolved the means to subvert defense signaling and breach PTI.

Plants are exposed to pathogens that have diverse infection strategies and therefore activation of appropriate, pathogen specific defense responses is vital for plant growth and productivity (Glazebrook, 2005). Structural alteration in cell wall including waxy cuticle layer, deposition of callose, suberin, and lignifications of cell wall provide protection contributing to non-host disease resistance (Dangl and Jones, 2001). Upon pathogen recognition, immediate early response genes including glutathione S transferase 6 (GST) and immediate early induced glucosyltransferase (IEGT) detoxify and protect cells from oxidative stress (Uquillas et al., 2004 and references therein). Salicylic acid (SA) dependent defense marker gene such as PR1 is induced later during pathogenesis involving the key signal transducer NPR1 (non-expressor of pathogenesis related genes 1) (Schenk et al., 2000). Specific response to pathogens is also mediated by gene-for-gene recognition leading to the activation of resistance (R) genes or recently termed effector-triggered immunity (ETI) in host (Nimchuk et al., 2003; Chisholm et al., 2006). Activation of R gene is usually accompanied by production of reactive oxygen species (ROS) leading to hypersensitive responses (HR) to restrict the spread of pathogen (Glazebrook, 2005). The localized cell death triggers systemic acquired resistance (SAR) to confer resistance throughout the plant (Baker et al., 1997). Hypersensitive response is also associated with induction of diverse group of defense related and pathogenesis related (PR) genes.

Plants defense responses are primarily associated with salicylic acid (SA), jasmonic acid (JA), and ethylene (ET) (Vlot et al., 2009). SA regulates the activation of defense responses against most biotrophic, hemi-biotrophic pathogens (Durner et al., 1997) and also mediates the establishment of systemic acquired resistance (SAR) (Grant and Lamb, 2006). By contrast, JA and ET operate synergistically to confer resistance against necrotrophic pathogens and herbivorous insects. JA is also associated with induced systemic resistance (ISR) elicited by rhizobacterial strains that promote plant growth and enhances resistance to various bacterial and fungal pathogens (Ton et al., 2002). In recent years, the role of other phytohormones including abscisic acid (ABA), auxins, gibberellins (GA), cytokines (CK), and brassinosteriods (BR) have started to emerge (Mutka et al., 2013) reviewed by Bari and Jones (2009). Apart from plant hormones, class of secondary metabolites including phenyl proponoid, glucosinolates, terpenoids, and phytoalexins aid in the protection of plant against most biotic stress (Kliebenstein, 2004). Synthesis and secretion of anti-microbial compounds confer selective advantage to curb pathogen invasion. Over 100,000 low-molecular-mass compounds derived from isoprenoid, polypropanoid, polyketide pathways are known to enhance defense against microbial infections (Dixon, 2001).

*Purple acid phosphatases* (*PAPs*) belong to a family of binuclear metalloenzymes and have been identified and characterized in numerous plants, animals, and a limited number of microorganisms (Mitic et al., 2006; Schenk et al., 2008). All PAPs contain dinuclear metal ions and a characteristic set of seven invariant residues, which coordinate the metal ions within the active site (reviewed by Mitic et al., 2006; Schenk et al., 2008). PAPs have been implicated in an array of biological functions. Most PAPs have been classified as non-specific acid phosphatases that catalyze the hydrolysis of inorganic phosphate (Pi) from various monoesters and anhydrides substrates (Olczak and Watorek, 2003). The physiological role of plant PAPs is predominantly associated with the regulation of Pi uptake and recycling (Li et al., 2002; Veljanovski et al., 2006). However, previous studies have also revealed roles for plant PAPs in other biological functions, including peroxidation (Del Pozo et al., 1999), ascorbate recycling (Zhang et al., 2008), mediation of salt tolerance (Liao et al., 2003), and regulation of cell wall carbohydrate biosynthesis (Kaida et al., 2009).

Recently, we demonstrated that the loss of *purple acid phosphatase5* (*PAP5*) in *Arabidopsis* leads to enhanced susceptibility to virulent *Pseudomonas syringae* pv. *tomato* DC3000 (*Pst* DC3000). *Arabidopsis* plants carrying a mutation in *PAP5* exhibited a defect in the expression of pathogenesis related (PR) genes including Pathogenesis Related gene 1 (*PR1*), Isochorismate synthase1 (*ICS1*) and plant defensin1.2 (*PDF1.2)*. This study also revealed that *pap5* plants failed to accumulate H_2_O_2_ in response to *Pst* DC3000 infection compared to wild-type plants (Ravichandran et al., 2013). One of the earliest responses to pathogen infection is generation of reactive oxygen intermediates ROIs (O^−^_2_ and H_2_O_2_). Although, ROIs such as hydroxyl radical (.OH), superoxide radical (O^−^_2_) and hydrogen peroxide (H_2_O_2_) are produced under normal metabolic processes. The rapid accumulation of ROS (also known as oxidative burst) cause oxidative cross linking of cell wall, activation of cellular signaling (protein phosphorylation) and induction of pathogenesis related (PR) genes (Alvarez et al., 1998). It is widely assumed that ROS production after pathogen recognition is associated with membrane bound NADPH oxidase in the apoplast (Lamb and Dixon, 1997). H_2_O_2_ generated in response to pathogen recognition induces salicylic acid (SA) and PR protein accumulation (Chamnongpol et al., 1998). A number of studies have indicated that ROS produced in response to pathogen recognition is located in the apoplast (reviewed by Torres et al., 2002). It is also evident that plants can produce ROS in other inter-cellular organelles including chloroplast, mitochondria and peroxisomes. However, the cellular homeostasis and concentration of ROS is highly regulated by enzymes such catalase, peroxidase and superoxide dismutase (Foyer and Noctor, 2003).

Having demonstrated that the loss of *PAP5* impaired plants innate immune responses (Ravichandran et al., 2013), we wanted to determine if overexpression of *PAP5* results in enhanced disease resistance. Further, *in silico* predictions failed to detect signal peptides on *PAP5*, hence we wanted to experimentally verify the prediction by tagging *PAP5* with a fluorescent label. Here, we report that the level of *PAP5* is crucial for mounting complete resistance against *Pst* DC3000. Optimal levels of *PAP5* are required for induction of PR genes and SA accumulation. Further, *PAP5* was found to be peroxisomal localized and aid the generation of reactive oxygen species for activation of defense responses.

## Results

### Optimal level of *PAP5* is required for complete resistance to Pst DC3000

**Figure 1:**
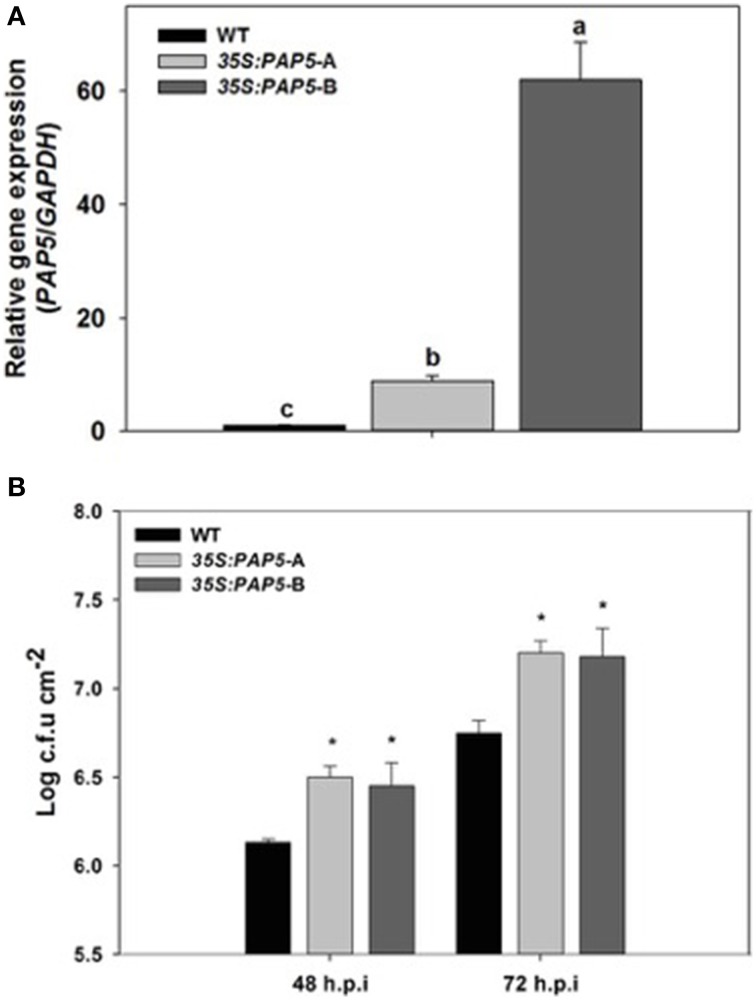
**Transgenic plants overexpressing ***PAP5*** exhibit enhanced disease susceptibility**. **(A)** Transcript levels of *PAP5* in transgenic plants. Total RNA was extracted from wild-type and transgenic plants as described in materials and methods. Transcript levels of *PAP5* was normalized to the expression of GAPDH in the same samples and expressed relative to the normalized transcript levels of wild-type plants. The bars represent the mean and standard deviation from two independent experiments. Significant differences (*P* < 0.05) are indicated by different letters. **(B)** Growth of *Pst* DC3000 in wild type and transgenic plants. Plants were inoculated with *Pst* DC3000 (10-8 c.f.u ml^−*l*^) and bacterial growth in plant apoplast was determined as described in the materials and methods. The bars represent the mean and standard deviation from values of three separate trials with six to eight replicates each trial. An asterisk indicates a significance increase in *Pst* DC3000 growth compared to wild-type plants (Student's *t*-test; *P* < 0.05).

Previously, we reported that loss of *PAP5* resulted in enhanced susceptibility of *Arabidopsis* to the virulent *Pst* DC3000 (Ravichandran et al., 2013). Having demonstrated that the loss of *PAP5* lead to enhanced susceptibility, we tested if overexpression of *PAP5* will result in enhanced resistance. We generated transgenic plants overexpressing *PAP5* under the control of constitutive cauliflower mosaic virus (CaMV) 35S promoter. Plants exhibiting Basta resistance were chosen and plants were picked randomly to verify the abundance of *PAP5* transcripts. Among the transgenic lines tested, two independent overexpressor lines *35S:PAP5*-A and *35S:PAP5*-B were chosen for further studies. Overexpressor lines *35S:PAP5*-A and *35S:PAP5*-B showed ~8 and 62-fold increase in *PAP5* transcripts respectively compared to wild-type (Col-0) plants (Figure 1A). There was no difference in the growth and development of both the overexpressing lines compared to wild-type plants.

For pathogenicity assay, plants were sprayed with suspension of *Pst* DC3000 as described in the methods. Interestingly, the overexpressor lines exhibited extensive chlorosis and increased susceptibility to *Pst* DC3000 compared to wild-type plants. Assessment of *Pst* DC3000 growth in plant apoplast revealed that both overexpressor lines had higher bacterial titers compared to wild-type plants (Figure 1B). Both overexpressor lines exhibited comparable levels of chlorosis and bacterial titers suggesting that the enhanced susceptibility is not due to the positional effects on insertion of the transgene. Although the overexpressor line *35S:PAP5*-B constitutively expressed higher levels (~62 fold) of *PAP5* compared to *35S:PAP5*-A plants (Figure 1A) we did not observe any significant difference in susceptibility to *Pst* DC3000 (Figure 1B).

### Overexpression of *PAP5* impairs pathogenesis related (PR) gene expression and alters H_2_O_2_ and salicylic acid accumulation

**Figure 2:**
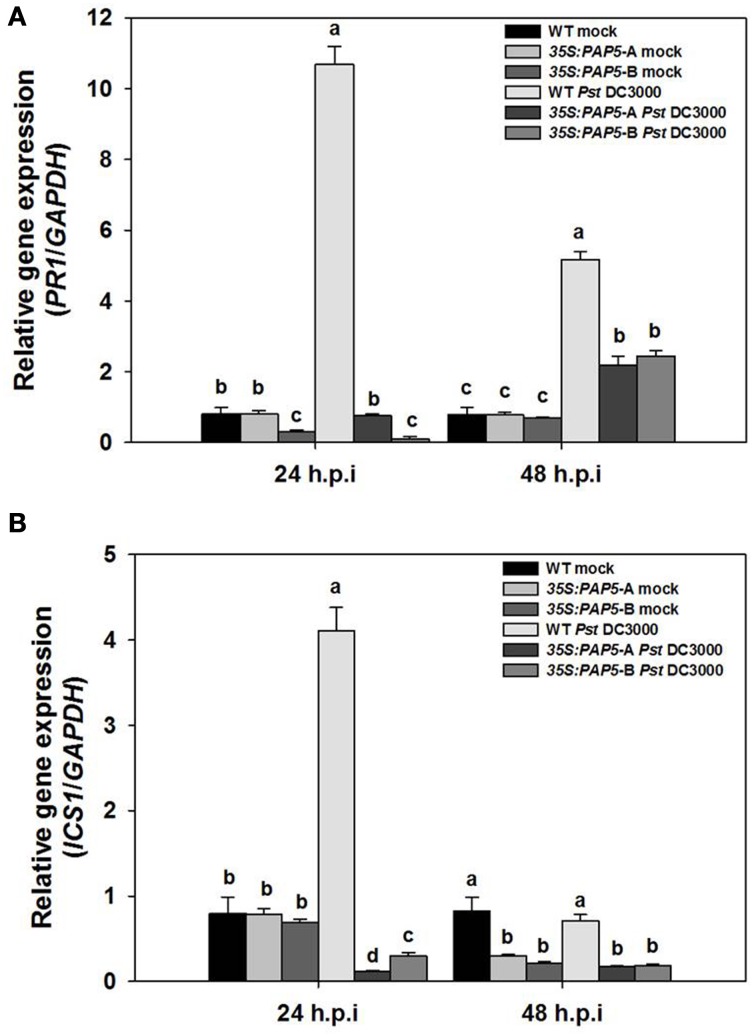
**Expression of ***ICS1*** and ***PR1*** is impaired in transgenic plants (***35S:PAP5***-A and ***35S:PAP5***-B)**. Plants were spray inoculated with *Pst* DC3000 (10^8^ c.f.u ml^−*l*^) and RNA was extracted from leaf tissues sampled 24 and 48 h.p.i. Transcript levels were normalized to the expression of *GAPDH* in the same samples. The transcript levels were expressed relative to the normalized transcript levels of mock infected wild-type plants. The bars represent the mean and standard deviation from two independent experiments. Transcript levels of *ICS1* **(A)** and *PR1* **(B)** in mock and *Pst* DC3000 infected plants. Significant differences (*P* < 0.05) are indicated by different letters (Student's *t*-test; *P* < 0.05).

In contrast to our expectation, overexpression of *PAP5* did not result in enhanced resistance. Therefore, we tested if expression of defense related genes are impaired in the overexpressor lines (*35:PAP5*-A and *35S:PAP5*-B). The overexpressor lines and wild-type plants were spray inoculated with suspension of *Pst* DC3000 (10^8^ c.f.u ml^−*l*^) and leaf tissues were harvested for gene expression analysis. The expression of pathogenesis related protein gene1 (*PR1*), a commonly used marker gene associated with *Pst* DC3000 infection and SA-mediated defense responses was observed. There was no significant difference in the transcript abundance of *PR1* between the mock infected overexpressor lines and the wild-type plants. However, *PR1* was strongly induced in infected wild-type plants 24 h.p.i., whereas it was not induced in OE lines (Figure 2A). Although the expression of *PR1* was slightly higher in both overexpression lines at 48 h.p.i., the levels of *PR1* was significantly lower compared to infected wild-type plants (Figure 2A). We also tested the expression of *isocorismate synthase1* (*ICS1*), which is responsible for ~90% of pathogen induced SA production (Wildermuth et al., 2002). As shown in Figure 2B, accumulation of *ICS1* was ~4 fold higher in infected wild-type plants, whereas the expression of *ICS1* was strongly suppressed in both the OE1, OE2 overexpressor lines. However, the expression of *ICS1* in infected wild-type plants was similar to mock infected wild-type plants at 48 h.p.i. Moreover, the expression of *ICS1* correlated with the expression of *PR1*.

**Figure 3:**
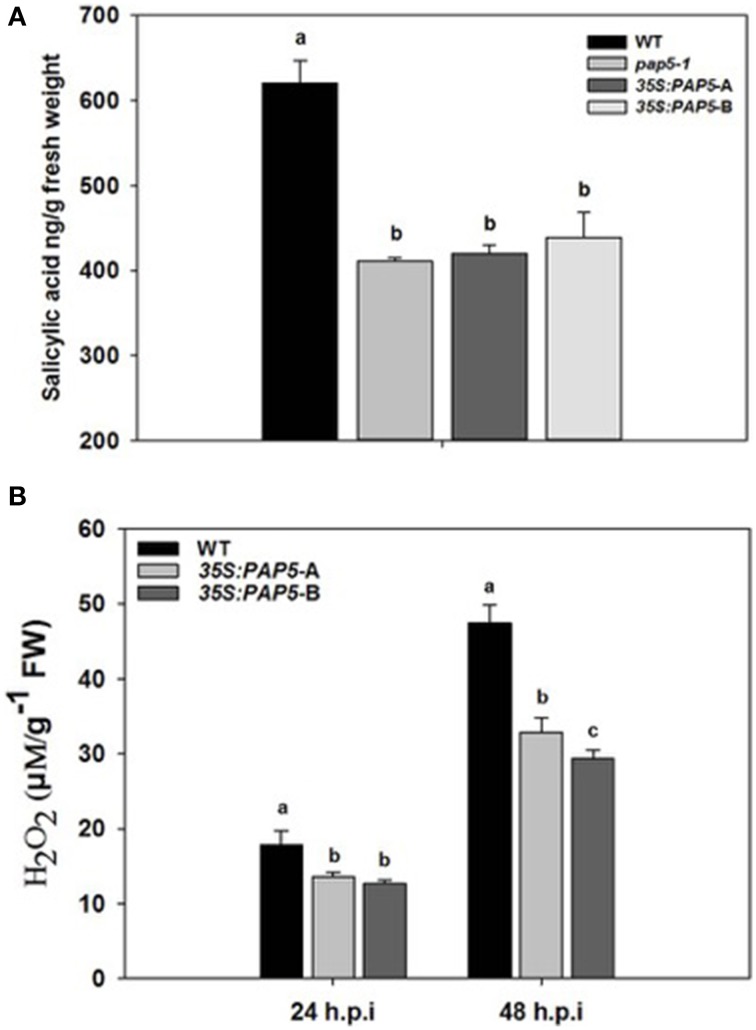
**Both loss and overexpression of ***PAP5*** affect salicylic acid (SA) and H_2_O_2_ accumulation in ***Pst*** DC3000 infected plants**. **(A)** Quantification of SA in *pap5-1* and 35S:*PAP5* plants following *Pst* DC3000 infection. Plants were spray inoculated with *Pst* DC3000 (10^8^ c.f.u ml^−*l*^) and leaf tissues were excised 48 h.p.i. The bars represent the mean and standard deviation from three replicates. Significant differences (*P* < 0.05) are indicated by different letters (Student's *t*-test; *P* < 0.05). **(B)** Quantification of H_2_O_2_ following Pst DC3000 infection. The bars represent mean and SD of H_2_O_2_ accumulation. A significant difference (*P* < 0.05) in H_2_O_2_ production are indicated by different letters (Student's t-test; *P* < 0.05).

To determine whether the decrease in the expression of ICS1 altered the concentration of salicylic acid (SA), we quantified the SA in the infected leaf tissue. We quantified SA in wild-type, overexpressor lines (*35:PAP5*-A and *35S:PAP5*-B) and knockout line *pap5-1* following *Pst* DC3000 infection. The concentration of SA increased in all *Pst* DC3000 infected plants. However, the SA levels in both the overexpressor lines and *pap5-1* plants were only ~60% of the wild-type plants (Figure 3A). In addition, accumulation of hydrogen peroxide (H_2_O_2_) generated in response *Pst* DC3000 was suppressed in both overexpressor lines (Figure 3B). These results suggest that the over expression of *PAP5* impaired the transcription of *ICS1, PR1*, and SA accumulation subsequently similar to that of the loss-of-function *pap5* mutant. Taken together, it is evident that optimal level of *PAP5* is required for expression of *ICS1* and *PR1* and for accumulation of H_2_O_2_ and SA after *Pst* DC3000 infection. Further, these results suggest that the enhanced growth of *Pst* DC3000 in *35S:PAP5*-A and *35S:PAP5*-B is dependent on SA accumulation and SA-mediated defense responses.

### Sub-cellular localization of *PAP5*

**Figure 4:**
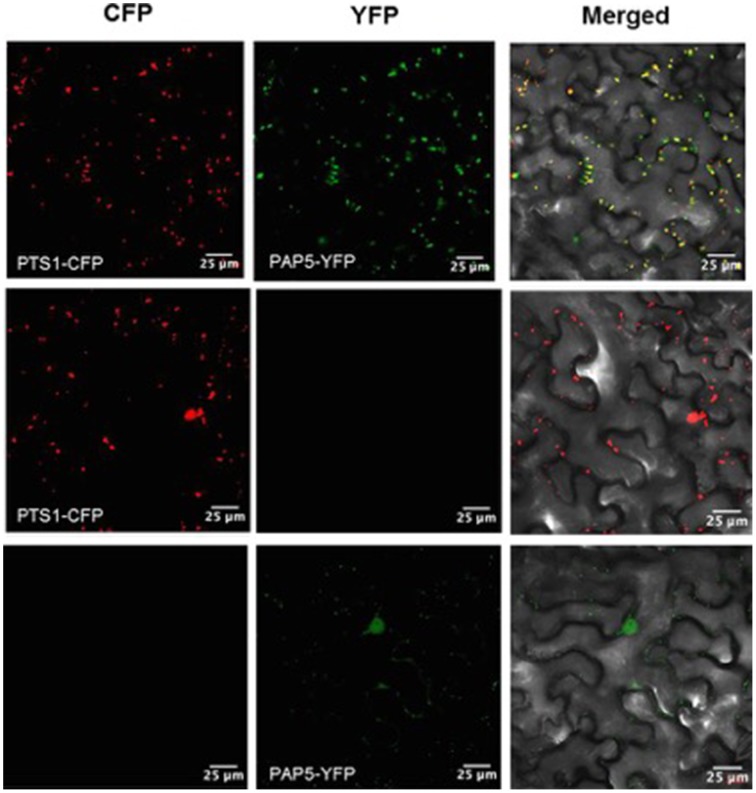
**Subcellular localization of ***PAP5*** in ***N. benthamiana*** leaves**. Agrobacterium strains carrying the recombinant plasmids (*35S:YFP-PAP5*) and peroxisomal specific marker (*PTS1:CFP*) was transiently expressed in tobacco leaves. Left plane shows a single optical section of CFP fluorescence images, the middle plane show single optical section of YFP fluorescence and right planes shows transmitted light images with merged CFP and YFP fluorescence. Bar = 25 μm.

To identify the sub-cellular localization of *PAP5*, a comprehensive *in silico* prediction was performed. Most *in silico* prediction revealed that *PAP5* could be localized to the nucleus, cytosol, extracellular, endoplasmic reticulum, golgi bodies and to the extracellular space (http://suba.plantenergy.uwa.edu.au/). However, a search on http://www.cbs.dtu.dk/services/SignalP/ revealed that *PAP5* does not carry a signal peptide. To verify this contradictory *in silico* prediction, the coding region of *PAP5* was fused to the YFP reporter gene under the control of CaMV 35S promoter. Confocal microscopy reveled *YFP-PAP5* as rapidly moving punctate structures within the cytoplasm and faintly in the nucleus (Figure 4). To identify the cellular compartment, the agrobacterium strains carrying organelle specific markers (Nelson et al., 2007) were coinfiltered with *YFP-PAP5* and leaf tissues were harvested at different time points for confocal microscopy. As shown in Figure 4, *YFP-PAP5* showed a strong colocalization with peroxisomal specific marker (peroxisomal targeting signal 1; *PTS1-CFP*). Agrobacterium strains carrying *YFP-PAP5* was also cofiltered with golgi (GmMan1 cytoplasmic tail and transmembrane) and plastid (rubisco targeting sequence) specific markers. In contrast to *in silico* predictions, *YFP-PAP5* did not localize to golgi or plastid organelle specific markers (Figure S1).

**Figure 5:**
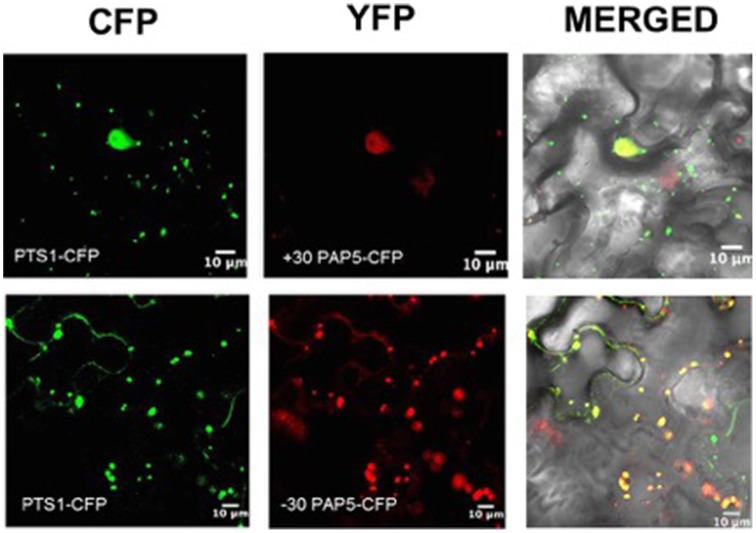
**Subcellular localization of ***PAP5*** in ***N. benthamiana*** leaves**. Agrobacterium strains carrying the recombinant plasmids (+*30PAP5:YFP or* +*30PAP5:YFP*) and peroxisomal specific marker (*PTS1-CFP*) was transiently expressed in tobacco leaves. Left plane shows a single optical section of CFP fluorescence images, middle planes show a single optical section of YFP fluorescence and right planes shows transmitted light images with merged CFP and YFP fluorescence. +*30PAP5:YFP* lacks a stretch of N-terminal amino acids MSLETFPPPA. −*30PAP5:YFP* lack N-terminal amino acids MSLETFPPPA. Bar = 10 μm.

Since the *in silico* prediction showed that *PAP5* lack signal peptides, we wanted to determine if removal of specific stretch of amino acids from C- or N-terminals affect the peroxisomal localization of *PAP5*. *PAP5* constructs lacking a stretch of N-terminal amino acids (MSLETFPPPA), referred as *YFP:+30PAP5* failed to localize to peroxisomes (Figure 5). However, removal of amino acids RYYLPEEETI from the C-terminal (referred as *YFP: -30PAP5*) of *PAP5* did not prevent the localization of *PAP5* to peroxisomes. These results suggest that N-terminal amino acids MSLETFPPPA are required for subcellular localization of *PAP5*.

## Discussion

In this study, we showed that an optimal level of *PAP5* is critical for mounting appropriate defense responses. Previous studies have revealed several molecular cues that regulate plant defense responses. Often, genes identified as positive regulator of defense responses are overexpressed to generate disease tolerant crops (Zhang et al., 2014). Interestingly, in some instances, both reduced and overexpression of a gene could result in the same phenotype. For example, *OXI1* (*Oxidative Signal-Inducible1*), encoding a serine/threonine kinase has been shown to be required for complete activation of mitogen-activated protein kinase (MAPKs). *oxi-1* null mutants showed enhanced susceptibility to virulent *Hyaloperonospora arabidopsidis* (formerly *Paranospora parasitica*) (Menke et al., 2004). Interestingly, transgenic plants overexpressing *OXI1* (*35S::OXI1*) displayed enhanced susceptibility to both virulent *Hyaloperonospora arabidopsidis* and *Pst* DC3000 (Petersen et al., 2009). Since both reduced and overexpression of *PAP5* lead to enhanced susceptibility to *Pst* DC3000, we hypothesize that *PAP5* could exist in complex with other proteins. Thus, constitutive expression of *PAP5* (*35S:PAP5*) alone may not be sufficient for complete resistance. It is also possible that the prolonged expression of *PAP5* could negatively affect basal resistance against *Pst* DC3000. Previously, we identified that *PAP5* is not expressed under normal growth conditions and are selectively induced only during prolonged Pi starvation and early stage of *Pst* DC3000 (6 h post inoculation) (Ravichandran et al., 2013). Hence, constitutive overexpression of *PAP5* is not optimal and may perturb and impair its function following *Pst* DC3000. Previous studies have also shown that both overexpression and partial loss of *FIP1* [FIN (Far-red insensitive) 219 Interacting Protein] resulted in hyposensitive hypocotyl phenotype under continuous Far Red (FR) light (Chen et al., 2007). *FIP1* was also shown to exhibit glutathione S-transferse activity which lead to delayed flowering phenotype under long-day conditions. Similarly, loss and overexpression of *EBS* (*Early Bolting in Short Days*) showed early flowering, a dwarf phenotype and reduced fertility (Pineiro et al., 2003). *EBS* encodes a nuclear protein with homeodomain Zn finger that regulate chromatin remodeling and repress the initiation of flowering in short days.

Pathogen recognition triggers generation of reactive oxygen intermediates (ROIs), which is required for activation of defense responses (Torres et al., 2002). It is also evident that the generation of ROI occurs within hours of pathogen infection (Alvarez et al., 1998). Since *PAP5* is induced only during the earlier stages (6 h.p.i) of *Pst* DC3000 infection and the localization of *PAP5* in peroxisome (Figure 4) suggests that *PAP5* may act as a component of ROI generation. Hence we hypothesized that *PAP5* might exist in complex with other proteins, a comprehensive *in silico* prediction was performed to identify proteins that may potentially interact with *PAP5*. Most *in silico* prediction searches on Bio-Analytic Resource for Plant Biology (BAR), Biological General Repository for Interaction Datasets (BioGRID) and GeneMANIA failed to detect any physical interaction. Moreover, most subcellular tools including SUBA, TargetP, and WoLF PSORT failed to identify the peroxisomal targeting sequences of *PAP5*. Previous studies have also shown that most *in silico* prediction programs fail to identify signature peroxisomal targeting sequences (Nelson et al., 2007). Few *in silico* prediction showed that *PAP5* is targeted to the extracellular space (http://suba.plantenergy.uwa.edu.au/; http://wolfpsort.org/).

In mammals, the high expression of PAPs in macrophages and increased ROS production that mediates microbial killing has been demonstrated (Kaija et al., 2002). Although ROS are produced under normal metabolic processes, the role of ROS in signaling is largely dependent on the rate of synthesis and is controlled by antioxidative enzymes such as catalase and peoxidases in peroxisomes (Nyathi and Baker, 2006). Previously, catalase deficient plants have been shown to display marked perturbation of intracellular redox and cellular homeostasis (Vandenabeele et al., 2004). Such perturbation is associated with accumulation of salicylic acid (SA) and induction of pathogenesis related (PR) genes (Chamnongpol et al., 1998). Similarly, catalase deficient *Arabidopsis* (*cat2*) plants showed increased peroxisomal H_2_O_2_ (Chaouch et al., 2010). Peroxisomal β-oxidation is also attributed to the degradation of various straight and branched chain fatty acids (Baker et al., 1997). Derivatives of β-oxidation such as cyclic oxylipins also play a significant role in the synthesis of plant hormones jasmonic acid (JA) and salicylic acid (SA) which are important signaling molecules (Theodoulou et al., 2005). Following *Pst* DC3000, infection the SA levels in both overexpressor lines and *pap5-1* plants were ~60% of the wild-type plants. These results also suggest that SA accumulation is not completely abolished in both transgenic (*35S:PAP5*) and *pap5-1* plants.

**Figure 6:**
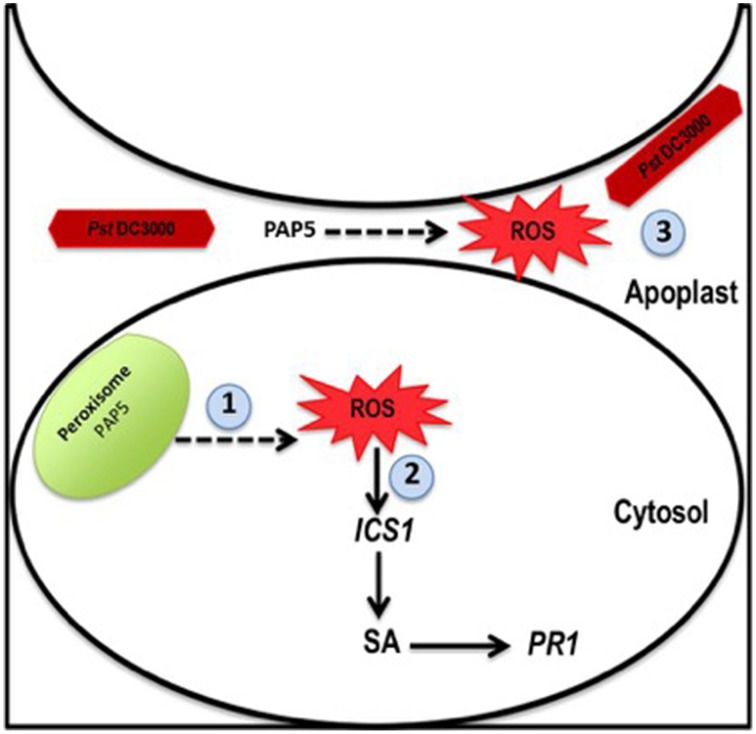
**Model for role of ***PAP5*** during ***Pst*** DC3000 infection**. When plants are infected with virulent *Pst* DC3000 1. Peroxisomal localized *PAP5* may be required for activation of glycolate oxidase, which modulate H_2_O_2_ generation. 2. ROS induces several defense responsive genes including *PR1* and *ICS1*. 3. ROS secreted to the apoplast may directly affect *Pst* DC3000. Recognition of *Pst* DC3000, induce expression of *PAP5* only during the early stages of infection (6 h) and triggers ROS synthesis which subsequently activates other defense related signals down stream for complete resistance.

It is well recognized that majority of the eukaryotic proteins undergo reversible phosphorylation via protein kinase (PK) and phosphatase (PP) to control major cellular processes. A large family of protein kinases has been characterized and their function in various cellular processes has been well established (País et al., 2009). However, the physiological role of its counter partner protein phosphates has been poorly understood. Activation of sucrose phosphate synthase (SPS) and nitrate reduction (NR) has been associated with decrease in phosphorylation status of SPE and NR (Huber and Huber, 1996). Interestingly, phosphorylation of Ser158 is sufficientfor inactivation of spinach SPS *in vitro* (Huber and Huber, 1992). Similarly, *PAP5* may be required for complete activation of vital enzymes such as glycolate oxidase in peroxisomes that modulate H_2_O_2_ generation (Figure 6). Since ROS is generated under normal metabolic processes, a highly regulated mechanism must exist to control ROS generation on pathogen recognition.

Several PAPs (SAP1, SAP2, AtPAP17, and AtPAP26) induced under Pi starvation are secreted to the extracellular space to hydrolyze Pi containing substrates and also exhibit peroxidase activity (Del Pozo et al., 1999; Bozzo et al., 2002; Hurley et al., 2010). However, the role of PAPs and its peroxidase activity in the extracellular space is not clear. Previously, PAPs exhibiting peroxidase activity has been speculated to function in ROS production similar to the oxidative burst that occurs in response to pathogen recognition (Bozzo et al., 2002; Hurley et al., 2010). Bacterial pathogens including *Pst* DC3000 reaches the apoplastic region to acquire nutrients (Alfano and Collmer, 1996). It is possible that the PAPs secreted to the extracellular space have dual functions, i.e., the hydrolytic activity under Pi starvation and microbial killing during pathogenesis. Hence, H_2_O_2_ generated in response to *Pst* DC3000 infection is necessary for activation of basal defense responses. Further, H_2_O_2_ secreted to the extracellular space and apoplast may restrict *Pst* DC3000 growth directly. Taken together, these evidences suggest that peroxisomal localized *PAP5* plays a vital role in basal defense response. Moreover, an optimal level of *PAP5* is critical for maintaining complete basal resistance during pathogenesis. It is evident that the isoform of PAP have evolved to attribute various biological functions in plants.

## Materials and methods

### Biological materials and growth conditions

*Arabidopsis thaliana* (L.) Heynh, ecotype Columbia (Col-0) seeds were purchased from Lehle seeds (Round Rock, TX, USA) and *pap5* T-DNA insertion mutant line was obtained from *Arabidopsis* Biological Resource Center (Columbus, OH, USA). Stratified seeds were planted on Jiffy peat pellets (Halifax seeds, Canada) and seedling were grown at 22 ± 2°C with a photoperiod of 16 h light at 125 μmol m^−2^s^−1^ and 8 h dark cycle. Virulent *Pseudomonas syringae* pv. *tomato* DC3000 (*Pst* DC3000) was a kind gift from Dr. Diane Cuppels, Agriculture and Agri Food Canada (AAFC), ON, Canada. *Pst* DC3000 strains was maintained on King's medium B supplemented with rifampicin (50 μg ml^−*l*^).

### Pathogen inoculation

For pathogenicity assay, 4–5 week old plants were spray inoculation with bacterial suspension of virulent *Pst* DC3000. Plant inoculation and bacterial growth in plant apoplast was determined as described earlier (Ravichandran et al., 2013). In brief, strains of *Pst* DC3000 was cultured in King's medium B supplemented with rifampicin (50 μg ml^−*l*^) at 28°C until OD_600_ of 0.8. Bacterial cells were collected by centrifugation and resuspended in water containing 0.02% Silwet L-77 (Lehle seeds, USA) to a final concentration of 10^8^ c.f.u ml^−*l*^. Plants were spray inoculated and kept under high humidity for disease development. Leaves were excised (8-10 replicates) from different infected plants and were surface sterilized with ethanol (75% v/v). Four to five samples were made by pooling 2 leaf discs (0.5 cm^2^) and the samples were ground in sterile water with microfuge tube pestle. The ground tissues were serially diluted and plated on King's B medium containing rifampicin (50 μg/ml). The plates were incubated at 28°C and colonies were counter after 48 h. For *Pst* DC3000 induced gene expression, plants were spray inoculated with bacterial suspension (10^8^ c.f.u ml^−*l*^) and leaf tissues were frozen in liquid nitrogen at the time points indicated.

### RNA extraction and quantitative real-time PCR

Total RNA was extracted from frozen leaf tissues (3 plants per replicate) for two biological replicates using a monophasic extraction method (Chomczynski and Sacchi, 1987). Total RNA was treated with DNase (Promega, WI, USA) and Reverse Transcription was performed with 1 μg of total RNA using High-Capacity cDNA Reverse Transcription kit (Applied Biosystems, ON, Canada). Relative transcript levels were assayed by Real-Time PCR using gene specific primers (Table 1) on a StepOnePlus Real-Time PCR system (Applied Biosystems, ON, Canada), using SYBR Green reagent (Applied Biosystems, ON, Canada). To determine relative expression levels, the amount of target gene (three technical replicates/sample) was normalized over the abundance of constitutive *GAPDH* as endogenous controls. Primers were generated spanning an intron if possible using Primer3Plus (http://www.bioinformatics.nl/cgi-bin/primer3plus/primer3plus.cgi/).

**Table 1:** **List of primer sequences used in RT-qPCR**.

**Gene**	**Locus**	**Primer sequences (5′–3′)**
*GAPDH*	At1g13440	TTGGTGACAACAGGTCAAGCAAAACTTGTCGCTCAATGCAATC
*ICS1*	At1g74710	GCGTCGTTCGGTTACAGGACAGCGAGGCTGAATATCAT
*PAP5*	At1g52940	AACAGGTCGCTCCACTAGACATGGTTAGAGGCATATGTTTGTCC
*PR1*	At2g14610	TGATCCTCGTGGGAATTATGTTGCATGATCACATCATTACTTCAT

### Quantification of salicylic acid (SA) and hydrogen peroxide (H_2_O_2_)

For SA and JA quantification 4–5 week plants were spray inoculated with *Pst* DC3000 (10^8^ c.f.u ml^−*l*^). Leaves were excised (five plants per sample) in triplicates at 48 h.p.i and were snap frozen in liquid nitrogen. Leaf tissues (250 mg) were ground with liquid nitrogen and extracted with 5–10 ml of MeOH-H_2_O-HOAc (90:9:1). After 15 min the samples were centrifuged at 12,000 g for 10 min and the supernatant was collected. The extraction procedure was repeated twice. The pooled supernatant was dried under steam of N_2_ and suspended in 5 ml of 0.05% HOAc in H_2_O-MeCN (85:15). The samples were then filtered through 0.4 5 μm filter and meanwhile the SampliQ SAX (Aligent technologies, USA) cartridge was conditioned with 2 ml of MeOH. The cartridge was then equilibrated with 5 ml of water. The filtered samples were loaded on to SampliQ SAX cartridge and were washed with 5 ml of 50 mM sodium acetate in 5% methanol. The interphase (IP) was removed with 5 ml of methanol. SA and JA were eluted with 5 ml of 2% formic acid in methanol and dried under N_2_.

After optimization of the liquid chromatography conditions and the tuning of the Orbitrap mass spec for negative mode ionization, a dilution series of salicylic acid (SA) were injected. The detection was performed in negative mode monitoring for the exact mass of the pseudomolecular ion [M-H]^−^ at 137 amu. The samples (500 μg/ml) were analyzed with the same optimized LC/MS method using a Agilent analytical C18 (3.5 μm, 2.1 × 100 mm) with a gradient elution from 5% ACN in water to 100% ACN using 0.1% formic acid in both solvents.

For H_2_O_2_ quantification, leaf tissues were harvested (4–5 plants per replicate) for 4 biological replicate and was frozen and ground in liquid nitrogen. To 50 mg of ground frozen tissue, 500 μl of phosphate buffer (50 mM, sodium phosphate, pH-7.4) was added. The samples were centrifuged and 50 μl of the aliquot was used for H_2_O_2_ quantification, using an Amplex red hydrogen peroxide/peroxidase assay kit (Molecular Probes, Life Technologies, Canada).

### Cloning and plant transformation

The clone of interest was obtained from ABRC and cloned to gateway compatible expression vectors (Earley et al., 2006) using LR Clonase II Gateway Technology (Invitrogen, ON, Canada). Briefly, the clone DQ459170, containing full length *PAP5* (At1g52940) cDNA was obtained from ABRC. The full length cDNA was amplified without the stop codon via polymerase chain reaction (PCR) using TaKaRA Ex Taq® Polymerase (Clontech, USA). The PCR primers were designed to contain *att*B sites to enable Gateway® technology compatible cloning (Gateway® Technology, Life Technologies, Canada). Shine-Dalgarno and Kozak consensus sequences were included between the *att*B1 site and the start codon to allow protein expression in *E. coli* and mammalian cells. The fusion constructs lacking either N/C-terminal of *PAP5* was generated via PCR. The *PAP5* gene lacking N-terminal amino acids MSLETFPPPA (*YFP:*+*30PAP5*) was generated using primers 5′-GGTTATAACGCTCCTGAACAAGTT-3′ (forward) and 5′-GGTTATAACGCTCCTGAACAAGTT-3′ (reverse). *PAP5* gene lacking C-terminal amino acids RYYLPEEETI was amplified using primers (*-30PAP5:YFP*) using primers 5′-ATGTCACTCGAAACATTTCCTC-3′ (forward) and 5′-ATTTTTCAACCAAATAGAGTCTGCA-3′. The PCR product was purified and introduced to pDONR™ 221 vector as per manufactures instruction to generate entry clones (Gateway® Technology, Life Technologies, Canada). The recombinant plasmids were sequenced using the M13 sequencing primers to confirm the insert position and orientation.

The entry clone containing the full length *PAP5* or was introduced to the expression vector pEarleyGate 104 (Earley et al., 2006) and pMDC32 (Curtis and Grossniklaus, 2003) using LR clonase (Gateway® Technology, Life Technologies) to generate *35S:YFP-PAP5* and 35S:*PAP5* fusion constructs, respectively. Similarly, the fusion constructs lacking either N/C-terminal of *PAP5* was introduced to the expression vector pEarleyGate 104. The recombinant plasmids were introduced in to *Agrobacterium* strain GV310 (pMB90) using the freeze thaw method (Weigel and Glazebrook, 2005). The *Agrobacterium* strain carrying the fusion construct was used to transform *Arabidopsis* plants by floral dip method (Clough and Bent, 1998) or infiltrated in to tobacco plants for subcellular localization studies (Sparkes et al., 2006). The floral dip inoculation medium contained 0.5X Murashige and Skoog medium with 5.0% sucrose and 0.05% Silwet (Lehle seeds, TX, USA). Plants were selected for Hygromycin and Basta resistance for pMDC and pEarley vectors, respectively.

### Transient protein expression and subcellular localization

For subcellular localization 6 week old *Nicotiana benthamiana* (tobacco) plants were used. Tobacco plants were grown at 22 ± 2°C with a photoperiod of 16 h light at 125 μmol m^−2^s^−1^ and 8 h dark cycle. Plant organelle specific markers were obtained from ABRC (Nelson et al., 2007) and the plasmids were transformed to *Agrobacterium* strain GV310 (pMB90) using the freeze thaw method (Weigel and Glazebrook, 2005). Agrobacterium strains carrying the recombinant plasmids were grown in liquid Luria-Bertani (LB) media supplemented with appropriate antibiotics. Cells were harvested by centrifugation (5500 g for 10 min) and resuspended in infiltration medium to OD_600_ of 0.8. The infiltration medium contained 0.5% glucose, 50 mM MES, 2 mM Na_3_PO_4_, 0.1 mM acetosyringone (Sparkes et al., 2006). For co-expression of different constructs *Agrobacterium* suspension was mixed in equal proportion and the *Agrobacterium* suspension mixtures were infiltrated to the tobacco leaves using a needleless syringe. The leaf samples were excised 48 h after infiltration and mounted on a microscope slide in water. The images were obtained (single optical sections and Z-stack) using a Zeiss LSM 510 META inverted confocal laser scanning microscope (Carl Zeiss MicroImaging GmbH). For CFP fluorescence, excitation wavelength of 458 nm was used and emissions were collected between 475, 471 and 525 nm. For YFP fluorescence, an excitation wavelength of 514 nm was used and emissions were 472 collected between 530 and 600 nm. The fluorescence images were processed using Zeiss LSM Image Browser (Carl Zeiss MicroImaging GmbH).

### Conflict of interest statement

The authors declare that the research was conducted in the absence of any commercial or financial relationships that could be construed as a potential conflict of interest.

## Supplementary material

Figure S1 **Subcellular localization of *PAP5* in *N. benthamiana* leaves**. Agrobacterium strains carrying the recombinant plasmids (*35S:YFP-PAP5*) and Golgi or plastid specific markers were transiently expressed in tobacco leaves. Left plane shows a single optical section of CFP fluorescence images, middle planes show a single optical section of YFP fluorescence and right planes shows transmitted light images with merged CFP and YFP fluorescence. Bar = 25 μm.
