# Supplementary figures and images for: Optimal level of purple acid phosphatase5 is required for maintaining complete resistance to Pseudomonas syringae
Source: Front Plant Sci. 2015 Aug 4;6:568. doi: 10.3389/fpls.2015.00568 (PMC4523723; doi:10.3389/fpls.2015.00568)

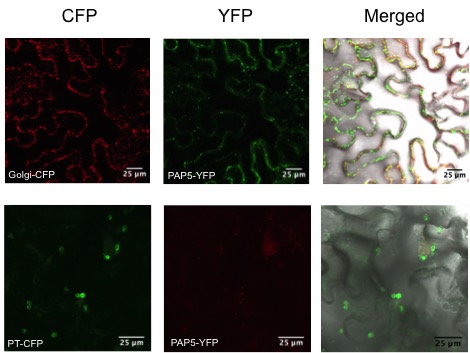

Supplement: Figure S1 — Subcellular localization of PAP5 in N. benthamiana leaves. Agrobacterium strains carrying the recombinant plasmids (35S:YFP-PAP5) and Golgi or plastid specific markers were transiently expressed in tobacco leaves. Left plane shows a single optical section of CFP fluorescence images, middle planes show a single optical section of YFP fluorescence and right planes shows transmitted light images with merged CFP and YFP fluorescence. Bar = 25 μm. [file Image1.JPEG]
